# *O*-GlcNAc stabilizes SMAD4 by inhibiting GSK-3β-mediated proteasomal degradation

**DOI:** 10.1038/s41598-020-76862-0

**Published:** 2020-11-16

**Authors:** Yeon Jung Kim, Min Jueng Kang, Eunah Kim, Tae Hyun Kweon, Yun Soo Park, Suena Ji, Won Ho Yang, Eugene C. Yi, Jin Won Cho

**Affiliations:** 1 Glycosylation Network Research Center, Yonsei University, 50 Yonsei-ro, Seodaemun-gu, Seoul, 03722 Republic of Korea; 2 Department of Systems Biology, College of Life Science and Biotechnology, Yonsei University, 50 Yonsei-ro, Seodaemun-gu, Seoul, 03722 Republic of Korea; 3 Interdisciplinary Program of Integrated OMICS for Biomedical Science, Graduate School, Yonsei University, 50 Yonsei-ro, Seodaemun-gu, Seoul, 03722 Republic of Korea; 4 Department of Molecular Medicine and Biopharmaceutical Sciences, Graduate School of Convergence Science and Technology and College of Medicine Or College of Pharmacy, Seoul National University, 28 Yeongeon-dong, Jongno-gu, Seoul, 03080 Republic of Korea

**Keywords:** Cell biology, Molecular biology

## Abstract

*O*-linked β-*N*-acetylglucosamine (*O*-GlcNAc) is a post-translational modification which occurs on the hydroxyl group of serine or threonine residues of nucleocytoplasmic proteins. It has been reported that the presence of this single sugar motif regulates various biological events by altering the fate of target proteins, such as their function, localization, and degradation. This study identified SMAD4 as a novel *O*-GlcNAc-modified protein. SMAD4 is a component of the SMAD transcriptional complex, a major regulator of the signaling pathway for the transforming growth factor-β (TGF-β). TGF-β is a powerful promoter of cancer EMT and metastasis. This study showed that the amount of SMAD4 proteins changes according to cellular *O*-GlcNAc levels in human lung cancer cells. This observation was made based on the prolonged half-life of SMAD4 proteins. The mechanism behind this interaction was that *O*-GlcNAc impeded interactions between SMAD4 and GSK-3β which promote proteasomal degradation of SMAD4. In addition, *O*-GlcNAc modification on SMAD4 Thr63 was responsible for stabilization. As a result, defects in *O*-GlcNAcylation on SMAD4 Thr63 attenuated the reporter activity of luciferase, the TGF-β-responsive SMAD binding element (SBE). This study’s findings imply that cellular O-GlcNAc may regulate the TGF-β/SMAD signaling pathway by stabilizing SMAD4.

## Introduction

TGF-β is a powerful inducer of cancer epithelial-mesenchymal transition (EMT) and metastasis^1^. Indeed, its expression of TGF-β is cause for a poor prognosis in the later stages of cancer^2,3^. The TGF-β signaling cascade starts with ligand binding to the type II TGFβ receptor, an auto-phosphorylated serine/threonine kinase^4^. The ligand-bound type II TGFβ receptor then recruits and trans-phosphorylates type I receptors^5,6^. Activated type I TGFβ receptors then phosphorylate the signaling effector component, SMAD2/3 (receptor-regulated SMADs, R-SMADs) on its c-terminal SXS motif^7,8^. Phosphorylated SMAD2/3 forms a heterotrimeric complex with co-operating SMAD4 (Co-SMADs)^9^, and the SMAD complex finally reaches the nucleus where it functions as a transcriptional factor in cooperation with other transcriptional factors and co-factors^10^.

In normal epithelium, TGF-β plays a tumor-suppressive role by arresting the cell cycle^11^. SMAD complexes regulate the transcription of the cyclin-dependent kinase inhibitors (CDKIs), p15^12^, and p21^13^. It also downregulates c-myc^14^ and ID family proteins^15^ involved in growth promotion. The well-established cytostatic effect of TGF-β is often lost during cancer progression^11^. Instead, it promotes cancer EMT, invasion, and metastasis as tumors develop by regulating EMT programming genes such as SNAIL1/2, TWIST 1/2, and ZEB1/2^16,17^. In addition to transcriptional reprogramming, TGF-β drives cancer EMT in a SMAD-independent way^18^ by changing the cytoskeleton and increasing cell junction dissolution^19^. This characteristic is usually referred to as ‘the TGF-β paradox’^3^. The fate of this signaling pathway is cell type- and context-dependent^2,20^. Owing to such multifunctionality and complexity, in-depth research on the TGF-β pathway is needed to develop targeted cancer therapy.

*O*-GlcNAc is a posttranslational modification that is produced in cytosol, the nucleus, and mitochondria^21–23^. Approximately 2–5% of glucose goes through the hexosamine biosynthetic pathway (HBP), and is converted into uridine 5′-diphospho-*N*-acetylglucosamine (UDP-GlcNAc), a donor substrate for *O*-GlcNAc transferase (OGT)^24,25^. OGT mediates the addition of sugar motif GlcNAc to hydroxyl groups of serine or threonine residues post-translationally whereas *O*-GlcNAcase (OGA) catalyzes the reversal of this modification^26^. *O*-GlcNAcylation can control a number of target proteins dynamically and reversibly by these two editing enzymes^27^. *O*-GlcNAc modification indicates cellular nutritional status^28^ because it utilizes UDP-GlcNAc, which is derived from diverse cellular metabolites, such as carbohydrates, amino acids, fatty acids, energy, and nucleotides^29,30^.

Hyperglycemia is regarded as an important risk factor for cancers^31^. It boosts HBP, which leads to aberrantly elevated *O*-GlcNAc levels. Increasing evidence shows that *O*-GlcNAc is a driving force for tumorigenesis^32^. A rise in cellular *O*-GlcNAc modification and upregulation of OGT are frequently found features of various cancers^33^ and reduced *O*-GlcNAc hinders cancer invasion and angiogenesis^34,35^. These observations led to this study of the functional role of *O*-GlcNAc in TGF-β-mediated signaling pathways. Both have key roles in cancer EMT but have never been linked together in cancer cells before. Furthermore, a recent study showed that the SMAD2/3 protein, a key effector of TGF-β, increased under hyperglycemic conditions^36^. Based on negative effect of SMAD4 deficiency on TGF-β signaling in breast cancer cells^37^, it was hypothesized in this study that the TGF-β signaling pathway was regulated by SMADs-*O*-GlcNAc axis.

This study examined SMAD4 as a new target for *O*-GlcNAc modification. *O*-GlcNAc stabilizes SMAD4 proteins by inhibiting their interaction with glycogen synthase kinase-3β (GSK-3β). *O*-GlcNAc on SMAD4 Thr63 competes with GSK-3β-driven *O*-phosphorylation which guides SMAD4 to be degraded^38^. Defects in *O*-GlcNAc modification on SMAD Thr63 antagonize TGF-β-responsive reporter gene activity in cancer cells. Consequently, this study’s results data suggest that *O*-GlcNAcylated SMAD4 is a new candidate for understanding how *O*-GlcNAc attributes to the TGF-β signaling cascade.

## Results

### Hyper-*O*-GlcNAcylation stabilizes SMAD4 proteins

**Figure 1:**
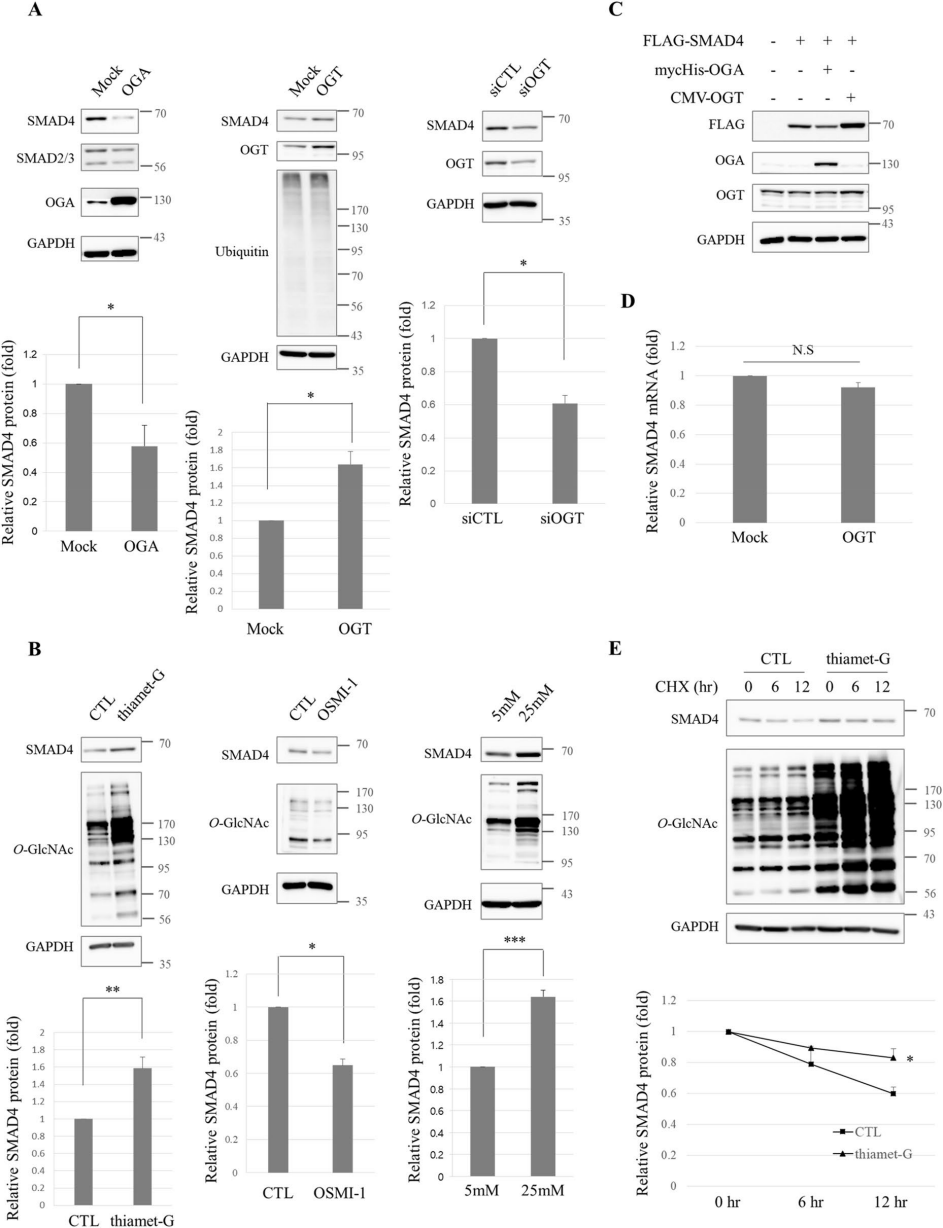
The stability of SMAD4 is associated with cellular *O*-GlcNAc levels in human lung cancer cell lines. (**A**) Immunoblots showing dropped endogenous SMAD4 in myc-His-OGA-overexpressing A549 cells (left panel). Cells were transfected with the expression vector encoding myc-His-OGA and harvested after 36 h. Immunoblot analyses representing elevated endogenous SMAD4 in the presence of pCMV-OGT (middle panel). The plasmid-encoding pCMV-OGT was transfected into A549 cells for 24 h. siRNA-mediated knock-down of OGT and decreased endogenous SMAD4 in A549 cells (right panel). Cells were treated with small interfering RNA-targeting OGT for 48 h. Data are given as mean ± standard error from at least three independent experiments. The significance was *p < 0.05. *P* values were calculated by Student’s *t* test. Full-length blots are presented in Supplementary Fig. S4. (**B**) Immunoblots representing growth in endogenous SMAD4 and *O*-GlcNAc levels in thiamet-G-treated A549 cells for 48 h (left panel). The culture medium was supplemented with 10 μM thiamet-G every 24 h. Immunoblot analyses indicating a decline in endogenous SMAD4 and *O*-GlcNAc levels in OSMI-1-incubated A549 cells (middle panel). Control cells were incubated with DMSO. Immunoblots showing increased endogenous SMAD4 and *O*-GlcNAc levels in A549 cells cultured in 25 mM hyperglycemia (right panel). Cells were grown in 5 mM normoglycemia and in 25 mM hyperglycemia for 48 h. Data are given as mean ± standard error from at least three independent experiments. The significances were *p < 0.05, **p < 0.01, and ***p < 0.001. *P* values were calculated by Student’s *t* test. Full-length blots are presented in Supplementary Fig. S4. (**C**) Immunoblots showing the regulation of exogenous FLAG-SMAD4 on pCMV-OGT or myc-His-OGA overexpression. The plasmids encoding FLAG-SMAD4 were co-transfected with pCMV-OGT or myc-His-OGA into A549 cells for 24 h. Full-length blots are presented in Supplementary Fig. S4. (**D**) No significant difference in mRNA levels of SMAD4 were observed as measured by quantitative RT-PCR. pCMV-OGT-overexpressing A549 cells were prepared after 24 h of transfection. GAPDH was used for normalization. Data are given as mean ± standard error (n = 3). Relative SMAD4 mRNA levels used for statistical analysis are presented in Supplementary Table S1. (**E**) Half-life of SMAD4 in the absence and presence of thiamet-G, respectively. Note that SMAD4 had a longer half-life after thiamet-G treatment (upper panel). A549 cells were treated with 10 μg/ml CHX for the indicated time period after incubation with 10 μM thiamet-G for 48 h and then harvested. SMAD4 protein levels (lower panel). Data are given as mean ± standard error (n = 3). The significance was *p < 0.05. *P* values were calculated by Student’s *t* test. Full-length blots are presented in Supplementary Fig. S4.

SMAD2/3 protein levels have been shown to significantly increase when rat cardiac fibroblasts were grown in high-glucose medium but *O*-GlcNAcase overexpression prevented this growth^36^. To test whether this result also occurred in cancer cells, several SMAD proteins in human lung cancer cell line A549 were examined in this study. Overexpressed OGA lowered the levels of all SMADs tested, SMAD4 most of all (Fig. 1A, left panel). Thus, SMAD4 protein levels were examined under various conditions that were hypothesized to affect the cellular *O*-GlcNAc modification state. SMAD4 protein levels rose when cellular *O*-GlcNAc levels rose by overexpressing OGT enzymes (Fig. 1A, middle panel). *O*-GlcNAcylation is an endogenous proteasomal inhibitor that interrupts ATPase activity of 26S proteasomes^39^. In other words, the quantity of most proteins can generally be regulated by *O*-GlcNAc status in a proteasome-dependent manner. In this study, proteasome activity was measured by blotting ubiquitin to answer this question, but no significant differences were found (Fig. 1A, middle panel). This result suggests that increases in hyper-*O*-GlcNAcylation-mediated SMAD4 protein levels did not occur as a result of an attenuated proteasomal functions. SMAD4 levels decreased when OGT was knocked down by siRNA (Fig. 1A, right panel). SMAD4 protein levels were checked by treating A549 lung cancer cells with inhibitors of *O*-GlcNAc editing enzymes which caused thiamet-G, an OGA inhibitor, to increase SMAD4 levels (Fig. 1B, left panel) whereas SMAD4 levels decreased when treated with OSMI-1, an OGT inhibitor (Fig. 1B, middle panel). Hyperglycemia, the physiological condition that is most likely to cause increases in global *O*-GlcNAc levels, raised SMAD4 levels (Fig. 1B, right panel). There were similar findings for exogenous FLAG-tagged SMAD4 as well. FLAG-SMAD4 levels decreased when OGA was co-expressed, but OGT co-expression increased SMAD4 levels (Fig. 1C). In conclusion, the amount of SMAD4 proteins varied according to overall *O*-GlcNAc status in human A549 lung cancer cells.

Then the mechanism by which cancer cells increase SMAD4 protein expression when global *O*-GlcNAc levels rise was examined. First SMAD4 mRNA levels were measured by quantitative RT-PCR when cellular *O*-GlcNAc levels were sufficiently raised by overexpressing OGT in A549 cells. The mRNA transcripts of the SMAD4 gene in A549 cells did not increase at all under induced hyper-*O*-GlcNAcylation (Fig. 1D). This result implies that *O*-GlcNAc modification changes SMAD4 protein levels by modulating protein stability. The half-life of SMAD4 was assessed by blocking new protein synthesis with cycloheximide (CHX). SMAD4 remained longer under thiamet-G-treated conditions than under the control conditions, which implied that hyper-*O*-GlcNAcylation increases the half-life of SMAD4 proteins (Fig. 1E). This evidence indicates that global *O*-GlcNAc levels affect proteasomal degradation of SMAD4 without affecting its mRNA levels.

### Interactions between SMAD4 and GSK-3β which lead to proteasomal degradation of SMAD4 are hindered by *O*-GlcNAc

Although *O*-GlcNAc was shown to disturb the degradation of SMAD4 proteins in A549 lung cancer cells, it was still unclear how *O*-GlcNAc interrupted SMAD4 degradation. This process had to be investigated in more detail at the molecular level. Phosphorylation has been reported to mark SMAD4 for proteasomal degradation. GSK-3β sequentially phosphorylates SMAD4 on Thr273, Thr269, and Thr265 residues and phosphodegron guides SMAD4 to be recognized by β-TrCP, a ubiquitin E3 ligase of SMAD4. As a consequence, SMAD4 is degraded by proteasomes^38^. *O*-GlcNAcylation takes place on the Ser or Thr residues of target proteins, as *O*-phosphorylation does^29^. Crosstalk has been reported to occur between *O*-GlcNAc and *O*-phosphorylation^40^, so it was hypothesized that the phosphorylation that drives SMAD4 degradation might be impeded by *O*-GlcNAc modification.

**Figure 2:**
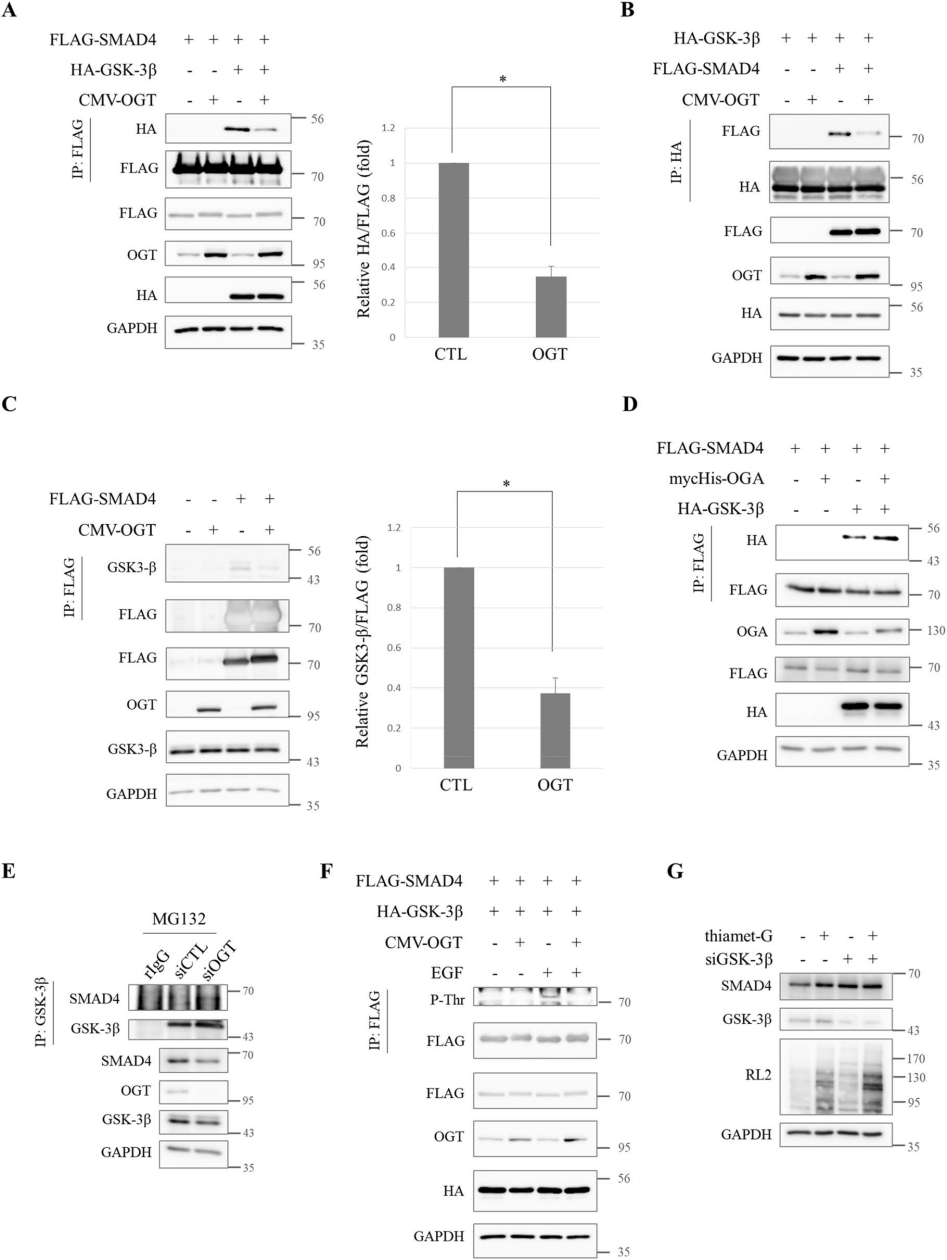
The interaction between SMAD4 and GSK-3β is interrupted by *O*-GlcNAc. (**A**) Immunoblots indicating hampered interactions between HA-GSK-3β and FLAG-SMAD4 when pCMV-OGT was overexpressed. Expression vectors encoding FLAG-SMAD4, HA-GSK-3β and pCMV-OGT were co-transfected in HEK293 cells for 24 h and whole lysates were subjected to immunoprecipitation using anti-FLAG. The precipitates were then immunoblotted with anti-HA to show the interactions between FLAG-SMAD4 and HA-GSK3β. Data are given as mean ± standard error (n = 3). The significance was *p < 0.05. *P* values were calculated by Student’s *t* test. Full-length blots are presented in Supplementary Fig. S4. (**B**) Western blot analyses showing alleviation of co-immunoprecipitated FLAG-SMAD4 in anti-HA-GSK-3β precipitates under pCMV-OGT-overexpressing conditions. HEK293 cells were transfected with plasmids encoding FLAG-SMAD4, HA-GSK-3β, and pCMV-OGT for 24 h and whole lysates were subjected to co-immunoprecipitation. Full-length blots are presented in Supplementary Fig. S4. (**C**) Immunoblots showing decreased endogenous GSK-3β interactions in anti-FLAG-SMAD4 immunoprecipitates. Constructs encoding FLAG-SMAD4 and pCMV-OGT were co-transfected into HEK293 cells for 24 h. An immunoprecipitation assay was then performed with anti-FLAG. The associated endogenous GSK-3β was detected by immunoblots using anti-GSK-3β. Data are given as mean ± standard error (n = 3). The significance was *p < 0.05. *P* values were calculated by Student’s *t* test. Full-length blots are presented in Supplementary Fig. S4. (**D**) Western blot analyses indicating increased interactions between FLAG-SMAD4 and HA-GSK3β when myc-His-OGA was overexpressed. HEK293 cells were transfected with plasmids encoding FLAG-SMAD4, HA-GSK-3β, and myc-His-OGA for 24 h and total lysates were subjected to co-immunoprecipitation using anti-FLAG. The precipitates were then immunoblotted with anti-HA to observe the interactions between FLAG-SMAD4 and HA-GSK-3β. Full-length blots are presented in Supplementary Fig. S4. (**E**) Immunoblots showing increased interactions between endogenous SMAD4 and GSK-3β when OGT was knocked down. A549 cells were treated with small interfering RNA-targeting OGT for 48 h and treated with 20 µM MG132 6 h prior to being harvested. An immunoprecipitation assay was then performed with anti-GSK-3β. The associated endogenous SMAD4 was detected by immunoblots using anti-SMAD4. Full-length blots are presented in Supplementary Fig. S4. (**F**) Immunoblot analyses showing reduced phospho-threonine levels of FLAG-SMAD4 in the presence of pCMV-OGT overexpression. HEK293 cells were transfected with plasmids encoding FLAG-SMAD4, HA-GSK-3β, and pCMV-OGT for 48 h. Cells were pre-treated with 100 ng/ml EGF for 24 h to enhance signaling. Full-length blots are presented in Supplementary Fig. S4. (**G**) Western blot data demonstrating no SMAD4 accumulation in GSK-3β k/d A549 cells. Cells were treated with small interfering RNA-targeting GSK-3β for 60 h and supplemented with 10 μM thiamet-G for 48 h. Full-length blots are presented in Supplementary Fig. S4.

In response to this hypothesis, several co-immunoprecipitation experiments were conducted to determine whether the interaction between SMAD4 and GSK-3β was affected by *O*-GlcNAc. Plasmids that encode FLAG-SMAD4 and HA-GSK-3β were co-transfected into HEK293 cells and total lysates were subjected to immunoprecipitation with anti-FLAG. The precipitates were blotted with anti-HA. The results showed that the interaction between FLAG-SMAD4 and HA-GSK-3β was weaker when OGT was overexpressed (Fig. 2A). The co-immunoprecipitation was repeated with HA-GSK-3β precipitates. Reduced levels of FLAG-SMAD4 were detected in HA-GSK-3β precipitates, indicating that the interactions between FLAG-SMAD4 and HA-GSK-3β were hampered under hyper-*O*-GlcNAcylation (Fig. 2B). Then the interaction between FLAG-SMAD4 and endogenous GSK-3β was examined. There was almost no interaction between FLAG-SMAD4 and endogenous GSK-3β when OGT was overexpressed (Fig. 2C). SMAD4 barely interacted with GSK-3β when global *O*-GlcNAc levels increased.

On the contrary, we examined the interaction between FLAG-SMAD4 and HA-GSK-3β when global *O*-GlcNAc status decreased in HEK293 cells. Increased levels of HA-GSK-3β were observed in FLAG-SMAD4 precipitates when OGA was overexpressed (Fig. 2D). We then checked the interaction between endogenous SMAD4 and GSK-3β in A549 lung cancer cells. A549 cells were treated with MG132 to prevent the degradation of SMAD4, which interacted with GSK-3β. We detected SMAD4 in GSK-3β precipitates when cellular *O*-GlcNAc levels decreased as a result of OGT knock-down (Fig. 2E). The interactions between SMAD4 and GSK-3β, which induces SMAD4 degradation, was shown to be negatively correlated with *O*-GlcNAc levels.

These observations led to the hypothesis that phospho-threonine of SMAD4 would be down-regulated by *O*-GlcNAc because of reduced interaction with GSK-3β. To determine whether this hypothesis was supported, immunoprecipitation was performed after HEK293 cells were transfected with expression vectors that encoded FLAG-SMAD4 and HA-GSK-3β. FLAG-SMAD4 precipitates were subject to immunoblotting with anti-phospho-threonine because there was no specific antibody against P-SMAD4 Thr265, Thr269, or Thr273 commercially available. Phospho-threonine levels of SMAD4 decreased when OGT was overexpressed (Fig. 2F). The intensity of anti-phospho-threonine was low, so signaling was strengthened via epidermal growth factor (EGF) treatment. EGF turns on ERK whose kinase activity facilitates phospho-SMAD4 T277 which in turn primes phosphorylation on Thr273, The269, and Thr265^38^. GSK-3β is an *O*-GlcNAc-modified protein^41^, so we checked whether OGT overexpression altered general GSK-3β activity. We measured GSK-3β activity by blotting HEK293 lysates with anti-phospho-GSK-3β Ser9 (inactive) and Tyr216 (active)^42^. Immunoblots indicated that there was no difference in GSK-3β activity when OGT was overexpressed (Supplementary Fig. S1). Taken together, these results showed that cellular *O*-GlcNAc stabilized SMAD4 by suppressing the GSK-3β-mediated phosphorylation that causes proteasome to degrade SMAD4 (Fig. 2). SMAD4 stabilization by hyper-*O*-GlcNAcylation did not occurred in GSK-3β k/d A549 cells (Fig. 2G). This result demonstrated that SMAD4 was stabilized by *O*-GlcNAc in GSK-3β-dependent manner.

### SMAD4 is modified by *O*-GlcNAc

**Figure 3:**
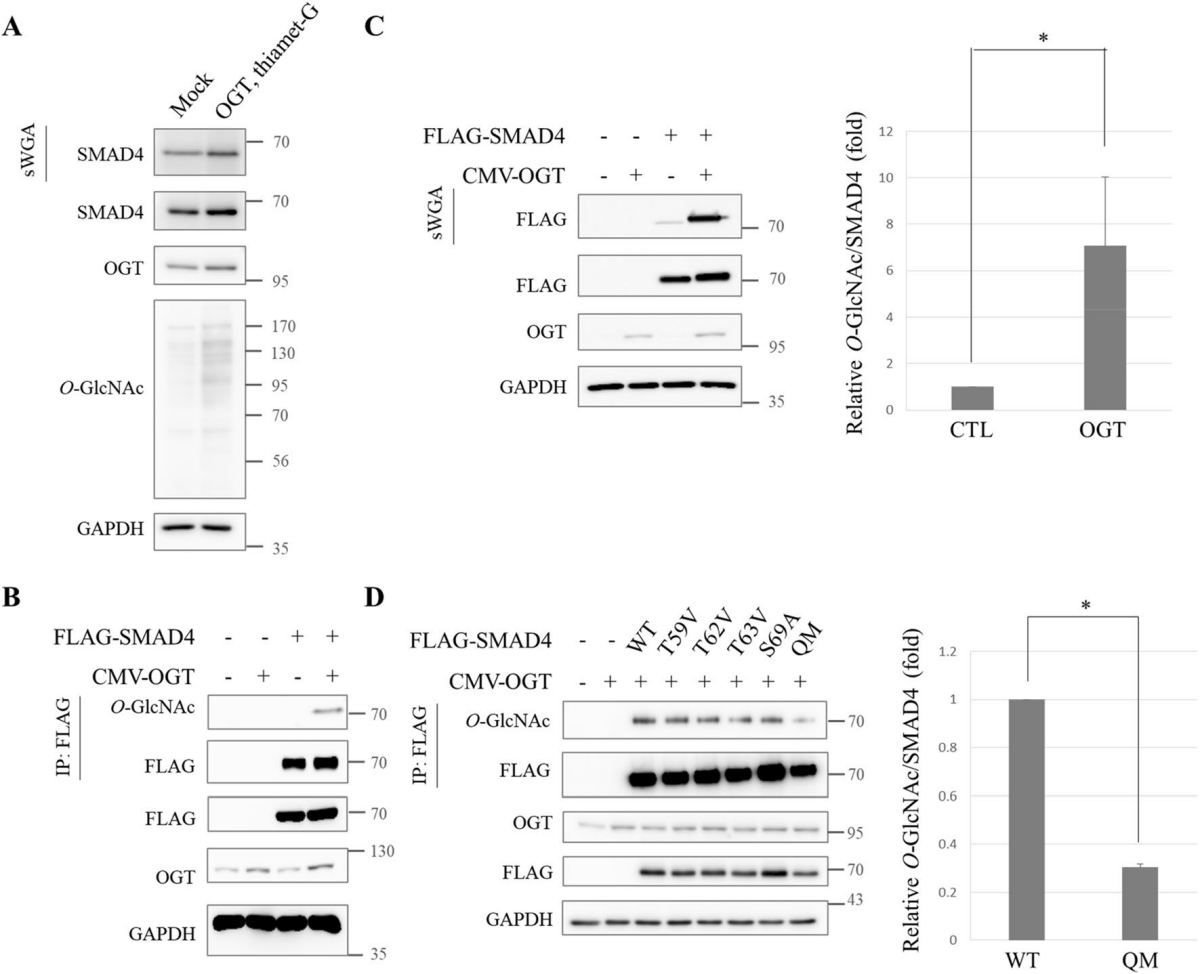
SMAD4 modified with *O*-GlcNAc at Thr59, Thr62, Thr63, and Ser69 residues. (**A**) *O*-GlcNAc on endogenous SMAD4. A549 cells were grown under thiamet-G-treated and pCMV-OGT-overexpressing conditions for 48 h. Whole cell lysates were then precipitated using sWGA. Full-length blots are presented in Supplementary Fig. S4. (**B**) Immunoblots showing *O*-GlcNAc of FLAG-SMAD4 and intensified modification by pCMV-OGT overexpression. HEK293 cells transiently expressing FLAG-SMAD4 and pCMV-OGT were prepared for immunoprecipitation with anti-FLAG. Immunoprecipitates were then probed with anti-*O*-GlcNAc. Full-length blots are presented in Supplementary Fig. S4. (**C**) A sWGA lectin affinity precipitation experiment showing *O*-GlcNAc on FLAG-SMAD4. Plasmids encoding FLAG-SMAD4 and pCMV-OGT were transfected into HEK293 cells for 24 h. Lectin precipitates were subjected to immunoblotting with anti-FLAG. Data are given as mean ± standard error (n = 3). The significance was *p < 0.05. *P* values were calculated by Student’s *t* test. Full-length blots are presented in Supplementary Fig. S4. (**D**) Immunoprecipitates showing decreased *O*-GlcNAc modification of the FLAG-SMAD4 QM. HEK293 cells were transfected with plasmids encoding FLAG-SMAD4 WT or five types of SMAD4 mutants for 24 h and then immunoprecipitated with anti-FLAG. Data are given as mean ± standard error (n = 3). The significance was *p < 0.05. *P* values were calculated by Student’s *t* test. Full-length blots are presented in Supplementary Fig. S4.

Then it was hypothesized that *O*-GlcNAc on SMAD4 might be responsible for inhibiting SMAD4′s interaction with GSK-3β. To test this hypothesis, first direct *O*-GlcNAc modification on SMAD4 was investigated. To examine whether endogenous SMAD4 carried *O*-GlcNAc modification in human cancer cells, succinylated wheat germ agglutinin (sWGA) was used for affinity purification of A549 whole cell lysates. The precipitates were probed with anti-SMAD4. Endogenous SMAD4 was shown to bear *O*-GlcNAc and the degree of modification increased when global *O*-GlcNAc was increased by OGT overexpression and thiamet-G treatment (Fig. 3A). The expression vector that encodes FLAG-SMAD4 was transiently overexpressed in HEK293 cells and immunoprecipitation was conducted with anti-FLAG. Immunoprecipitates were then subject to immunoblotting with anti-*O*-GlcNAc. FLAG-SMAD4 was *O*-GlcNAc-modified. The modification increased significantly when OGT was overexpressed (Fig. 3B). Lectin precipitation using sWGA verified FLAG-SMAD4′s *O*-GlcNAc modification, which was increased by OGT overexpression (Fig. 3C).

*O*-GlcNAc was identified on SMAD4 (Fig. 3A-C). Mass spectrometry analysis was conducted to narrow down the possible locations of *O*-GlcNAc modification within the SMAD4 protein. FLAG-SMAD4 was immunoprecipitated and subject to SDS-PAGE after preparing FLAG-SMAD4-overexpressing HEK293 cells and inducing OGT overexpression. The band of overexpressed FLAG-SMAD4 was then digested in-gel with both trypsin and chymotrypsin and analyzed. Thr59, Thr62, Thr63, and Ser69 residues were demonstrated to have *O*-GlcNAcylation (Supplementary Fig. S2). It had to be confirmed whether these amino acids possessed *O*-GlcNAc modification. Four single-point mutations and a quadruple mutation (QM) construct of SMAD4 were made by substituting threonine for valine and serine for alanine. They were ectopically expressed in HEK293 cells. Immunoprecipitation was conducted with all of the mutants. The QM displayed less *O*-GlcNAc modification, demonstrating that Thr59, Thr62, Thr63, and Ser69 were *O*-GlcNAcylation sites (Fig. 3D).

### *O*-GlcNAc on Thr63 is responsible for stabilizing SMAD4

**Figure 4:**
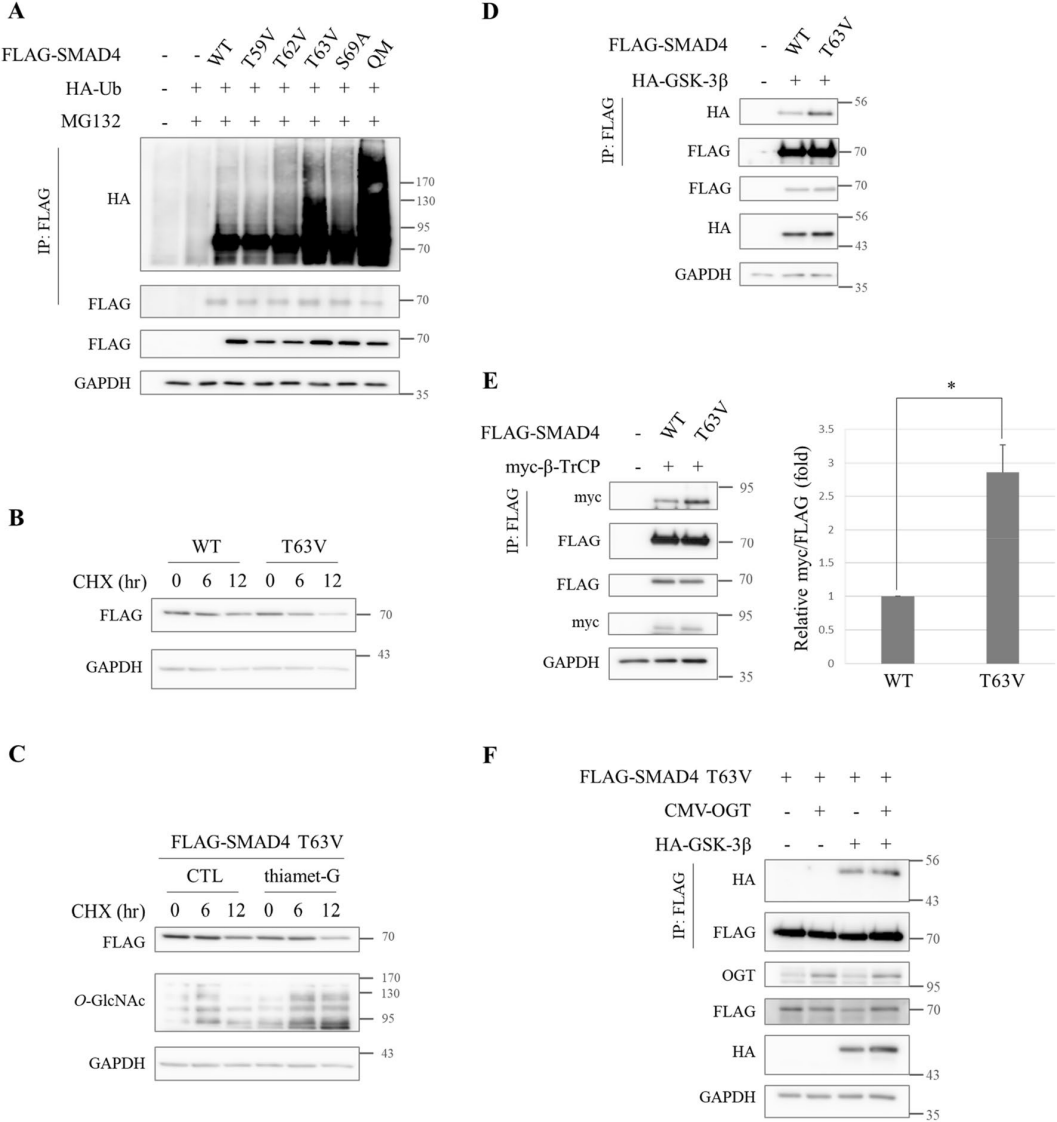
*O*-GlcNAc on SMAD4 T63 plays key role in SMAD4 stabilization. (**A**) Immunoblots demonstrating markedly increased ubiquitination of both FLAG-SMAD4 T63V and QM in comparison to WT. HA-Ub and FLAG-SMAD4 WT or mutant-expressing HEK293 cells were treated with 20 µM MG132 6 h prior to being harvested. Whole cell lysates were immunoprecipitated under 2% SDS-containing denaturation conditions with anti-FLAG. The level of SMAD4 ubiquitination was measured by immunoblotting precipitates with anti-HA. Full-length blots are presented in Supplementary Fig. S4. (**B**) The half-life of ectopic FLAG-SMAD4 WT and T63V mutants. FLAG-SMAD4 T63V mutants had a shortened half-life. A549 cells expressing FLAG-SMAD4 WT or FLAG-SMAD4 T63V were treated with 10 μg/ml CHX and harvested at the indicated time. Full-length blots are presented in Supplementary Fig. S4. (**C**) Western blot analyses verifying that SMAD4 T63V was resistant to stabilization by thiamet-G treatment. A549 cells expressing ectopic FLAG-SMAD4 T63V were treated with 10 μg/ml CHX at the indicated times after incubation with thiamet-G for 48 h. Full-length blots are presented in Supplementary Fig. S4. (**D**) Western blot analyses showing elevated interactions between FLAG-SMAD4 T63V mutants and HA-GSK-3β. Total lysates from Hek293 cells transfected with plasmids encoding FLAG-SMAD4 T63V and HA-GSK-3β were immunoprecipitated with anti-FLAG. Associated HA-GSK-3β bands were shown by immunoblotting precipitates with anti-HA. Full-length blots are presented in Supplementary Fig. S4. (**E**) Immunoblots demonstrating increased interactions between FLAG-SMAD4 T63V and E3 ligase myc-β-TrCP. Hek293 cells expressing FLAG-SMAD4 T63V and myc-β-TrCP were subjected to co-immunoprecipitation after 24 h of transfection. Interacting myc-β-TrCP was detected by immunoblotting precipitates with anti-myc. Data are given as mean ± standard error (n = 3). The significance was *p < 0.05. *P* values were calculated by Student’s *t* test. Full-length blots are presented in Supplementary Fig. S4. (**F**) Western blot analyses showing no differences in the level of interaction between FLAG-SMAD4 T63V and HA-GSK-3β under OGT overexpression. Expression vectors encoding FLAG-SMAD4 T63V, HA-GSK-3β and pCMV-OGT were co-transfected in HEK293 cells for 24 h and whole lysates were subjected to immunoprecipitation using anti-FLAG. The precipitates were then immunoblotted with anti-HA to show interactions between FLAG-SMAD4 T63V and HA-GSK-3β. Full-length blots are presented in Supplementary Fig. S4.

Then it was investigated whether *O*-GlcNAc on SMAD4 Thr59, Thr62, Thr63, and Ser69 stabilized SMAD4 by hindering its interaction with GSK-3β. Polyubiquitination plays a key role in the protein stability of SMAD4^43^. To better understand the critical *O*-GlcNAcylation residues that account for SMAD4 stabilization, the ubiquitination levels of the several SMAD4 substitution mutants were analyzed. HEK293 cells transiently co-expressing HA-Ub and series of SMAD4 substitution mutants were immunoprecipitated under denaturing conditions to avoid detecting the ubiquitination of proteins interacting with SMAD4. T63V and QM, mimicking hypo-*O*-GlcNAcylated SMAD4, facilitated SMAD4 polyubiquitination (Fig. 4A). This result underlines the importance of the *O*-GlcNAc modification in SMAD4 stabilization and shows that Thr63 is the critical residue.

Next, the half-life of SMAD4 WT was compared to that of the SMAD4 T63V mutant. The protein amounts were traced in a time-dependent manner in cells ectopically expressing FLAG-SMAD4 WT or FLAG-SMAD4 T63V. FLAG-SMAD4 T63V displayed accelerated degradation whereas FLAG-SMAD4 WT retained its half-life (Fig. 4B). Moreover, FLAG-SMAD4 T63V was resistant to being stabilized by thiamet-G unlike endogenous SMAD4 (Figs. 4C, 1E). These results demonstrated that *O*-GlcNAc on SMAD4 Thr63 protects against its proteasomal degradation. Interactions between the *O*-GlcNAc defective mutant FLAG-SMAD4 T63V and both HA-GSK-3β (Fig. 4D) and its E3 ligase myc-β-TrCP (Fig. 4E) dramatically increased. In addition, the interaction between FLAG-SMAD4 T63V and HA-GSK-3β was not affected by OGT overexpression, unlike FLAG-SMAD4 WT (Figs. 4F, 2A). These findings suggest that *O*-GlcNAc on SMAD4 Thr63 interrupts SMAD4′s interaction with GSK-3β, reducing the opportunities for hyper-*O*-GlcNAcylated SMAD4 to be recognized by β-TrCP, ultimately leading to SMAD4 stabilization.

### Defects in SMAD4 *O*-GlcNAc on Thr63 reduce responsiveness to TGF-β

**Figure 5:**
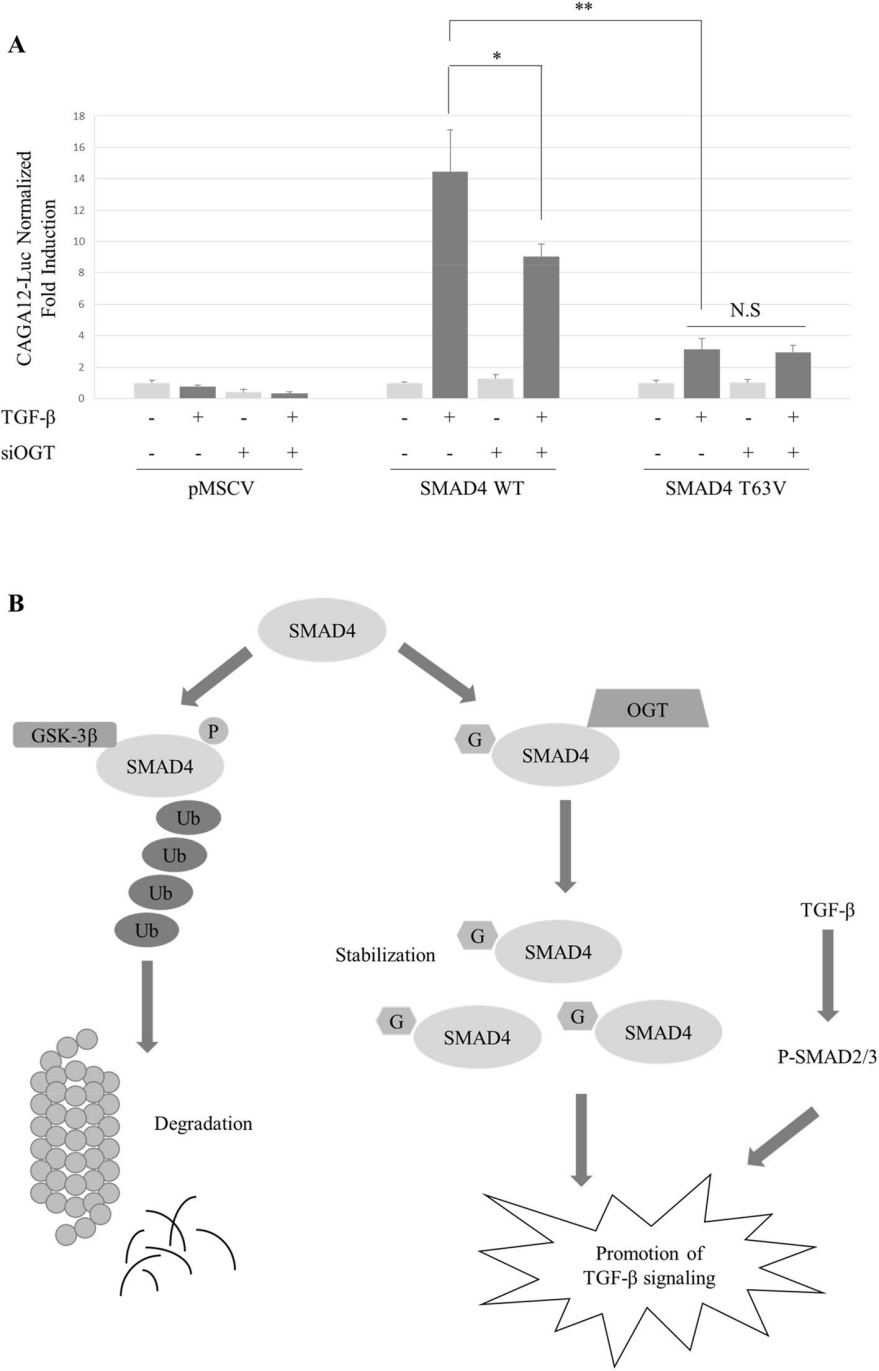
Defects in *O*-GlcNAc modification on SMAD4 T63 antagonize TGF-β signaling. (**A**) SBE reporter gene activity was downregulated in cells expressing SMAD4 T63V. MDA-MB-468 cells stably overexpressing SMAD4 WT or T63V were co-transfected with (CAGA)_12_-Luc and CMV-Renilla and incubated with siOGT for 48 h. 5 ng/ml TGF-β was introduced for 24 h. Data are given as mean ± standard error (n = 3). The significance were annotated as *p < 0.05 and **p < 0.01. *P* values were calculated using Student’s *t* test. Relative reporter gene activities used for statistical analysis are presented in Supplementary Table S2. (**B**) Proposed model for the contribution of *O*-GlcNAc in the TGF-β signaling pathway by stabilizing SMAD4 proteins. *O*-GlcNAc modification on SMAD4 T63 inhibits SMAD4’s interaction with GSK-3β which promotes SMAD4 degradation by proteasomes. As a result, defects in SMAD4 T63 *O*-GlcNAc attenuated TGF-β-responsive SBE reporter gene activity.

SMAD4 is a master regulator of the TGF-β signaling pathway. TGF-β prompts cancer cells to induce EMT and metastasis^44^. Then it was investigated whether *O*-GlcNAc modification affects TGF-β-responsive SBE luciferase reporter activity. SMAD4 WT and T63V mutants were cloned into the retroviral vector pMSCV-FLAG, followed by viral infection with MDA-MB-468, a SMAD4-null breast cancer cell line (Supplementary Fig. S3). Knock-down of OGT diminished reporter gene activity in puromycin-selected MDA-MB-468 cells expressing SMAD4 WT (Fig. 5A). The reporter gene activity of SMAD4 T63V was significantly lower than that of SMAD4 WT. Furthermore, the responsiveness of SMAD4 T63V towards TGF-β was not sensitive to OGT knock-down. Collectively, these findings indicate that *O*-GlcNAc plays a functional role in the TGF-β-induced signaling pathway by stabilizing SMAD4 (Fig. 5B).

## Discussion

Emerging evidence has shown that elevated *O*-GlcNAcylation is a hallmark of cancer and contributes to tumor progression^33,45^. Cancer cells have greater uptake of glucose and glutamine than normal cells^46^. *O*-GlcNAc deficiency reduces transformed cancer phenotypes in breast and prostate cancer cells^34,35^. Reduction in OGT decreases proliferation, invasive phenotypes, and angiogenic potential in prostatic cancer cells^34^ and inhibits cell growth and invasion in breast cancer cells^35^. Based on these findings, a search was made for another target of *O*-GlcNAc transferase that participates in carcinogenesis. Interestingly, A549 lung cancer cells increase glucose uptake, *O*-GlcNAc status, and OGT expression during EMT induced by TGF-β^47^, so we assumed that there would be a new *O*-GlcNAc-modified protein delivering TGF-β signals. In addition, the protein levels of SMAD2/3, major regulators of this signaling pathway, increase under hyperglycemia^36^. This research was conducted to investigate whether *O*-GlcNAc modulates TGF-β signaling pathway.

SMAD4 protein levels were demonstrated to correlate with global *O*-GlcNAc status (Fig. 1A,B). This result was consistent with a study which demonstrated that SMAD2/3 increases under hyperglycemic conditions in cardiac fibroblasts^36^. Increments of SMAD4 were found to have come from the presence of *O*-GlcNAc on SMAD4 Thr63 (Fig. 4). A number of proteins are directly stabilized by *O*-GlcNAc modification, such as RACK1^48^, Bmi-1^49^, and BMAL1/CLOCK^50^, whereas FOXM1^35^ and HIF1α^51^ are indirectly stabilized. Cellular *O*-GlcNAcylation also stabilizes proteins by inhibiting proteasome activity^39^, so ubiquitin blots were conducted to confirm that SMAD4 stabilization was not a product of proteasome inhibition (Fig. 1A, middle panel). The step had to be distinguished from the one in which SMAD4 stabilization originated. It is worth examining whether hyperglycemia-mediated SMAD2/3 elevation^36^ is a product of *O*-GlcNAc-induced protein stabilization.

There are numerous *O*-GlcNAc-modified proteins that participate in oncogenesis. EMT regulator SNAIL1^52^, tumor suppressor p53^53^, proto-oncogene β-catenin^54^, and c-myc^55^ are the *O*-GlcNAcylated proteins. In many cases, *O*-GlcNAc regulates target proteins by altering their crucial *O*-phosphorylation states^40^. For the first time in this study, it was revealed that SMAD4, a major regulator of TGF-β signaling preceding cancer EMT and metastasis in advanced cancers, was a target for *O*-GlcNAcylation (Fig. 3) and that *O*-phosphorylation is interrupted during hyper-*O*-GlcNAcylation caused by OGT overexpression (Fig. 2F). It would be interesting to study the effect of *O*-GlcNAc on other cancer transformation-related proteins that are affected by *O*-phosphorylation.

Surprisingly, SMAD4^38^, SNAIL1^52,56^, and β-catenin^57,58^, whose stabilities are lengthened by *O*-GlcNAc, employ the same E3 ligase, β-TrCP, for polyubiquitination. In addition, *O*-phosphorylation by GSK-3β guides all of them to be recognized by β-TrCP. Co-immunoprecipitation experiments using SMAD4 T63V mutants revealed that hampered interaction between SMAD4 and either GSK-3β or β-TrCP resulted from *O*-GlcNAc on SMAD4 (Fig. 4D, E). However, there can be a synergistic effect with *O*-GlcNAc to reduce interactions of GSK-3β with SMAD4 because GSK-3β also carries *O*-GlcNAc^41^. Accordingly, it would be interesting to further investigate whether *O*-GlcNAc-modified GSK-3β loses its binding affinity towards substrates.

A decrease in SMAD4 expression diminishes TGF-β-stimulated EMT or metastasis in several cancer cells^37,59^. Reporter gene activity of cells expressing SMAD4 T63V suppressed TGF-β signaling by destabilizing SMAD4 proteins (Fig. 5A). It needs to be further investigated whether defects in SMAD4 *O*-GlcNAc antagonize TGF-β-induced EMT, tumor migration, or invasion in various cancer cells. The TGF-β signaling cascade is challenging to study because it has dual functions that change depending on cancer stage and cell context^2,20^. This study verified that SMAD4 protein was stabilized by *O*-GlcNAc on Thr63. Even though the QM displayed hypo-*O*-GlcNAcylated SMAD4, not every mutant showed decline in their *O*-GlcNAc levels (Fig. 3D). There may be a compensational mechanism because four residues for *O*-GlcNAc modification were located nearby. Remaining issues regarding *O*-GlcNAc on Thr59, Thr62, and Ser69 also should be addressed to fully understand how the TGF-β signaling pathway is regulated by the SMAD4/*O*-GlcNAc axis.

## Methods

### Cell lines and reagents

A549, HEK293, and MDA-MB-468 cells were purchased from ATCC and HEK293FT cells were purchased from Thermo. Cells were cultured in Dulbecco’s Modified Eagle’s Medium (Lonza) supplemented with 10% fetal bovine serum, 100 μg/ml streptomycin, and 100 U/ml penicillin at 37 °C in 5% CO_2_.

Cells were treated with 10 μM thiamet-G which was kindly provided by Prof. Injae Shin (Yonsei University, Seoul, South Korea). 10 μg/ml Cycloheximide and 20 μM MG132 were purchased from Sigma. TGF-β was purchased from R & D systems.

### DNA transfection and RNA interference

DNA plasmids and siRNA were transfected into the cells with Lipofectamine 2000 (Thermo) or Lipofectamine RNAiMAX (Thermo) reagents following the manufacturer’s instructions. The specific custom siRNAs used in this study were as follows:

- siOGT sense, 5′-UAAUCAUUUCAAUAACUGCUUCUGC (dTdT)-3′
- siOGT antisense, 5′-GCAGAAGCAGUUAUUGAAAUGAUUA (dTdT)-3′
- siGSK-3β sense, 5′- GUCGCCAUCAAGAAAGUAU (dTdT)-3′
- siGSK-3β antisense, 5′- AUACUUUCUUGAUGGCGAC (dTdT)-3′

### Retrovirus infection

SMAD4 WT and T63V were cloned into the retroviral vector pMSCV-FLAG to induce stable expression. To obtain viral particles, retroviral packaging plasmids pVSV-G and Gag/Pol constructs were co-transfected into HEK293FT cells using Lipofectamine 2000 (Thermo). 48 h after transfection, the retroviral particles were collected and filtered. Supernatants were then infected in target MDA-MB-468 cells with 10 μg/ml final concentration of polybrene. Virally infected MDA-MB-468 cells were selected with puromycin.

### RNA isolation and quantitative RT-PCR

Total RNA was isolated using TRIzol reagent (Invitrogen). Total mRNA was reverse transcribed into complementary DNA using ReverTra Ace qPCR RT Master Mix (Toyobo). Quantitative real-time PCR was conducted using SYBR Premix Ex Taq (Takara) and analyzed in the Applied Biosystems 7300 Real-time PCR system. Gene-specific primers were as follows:

- SMAD4, forward 5′- TGCCTCACCACCAAAACGG-3′,
- SMAD4, reverse 5′- CCAAACAAAAGCGATCTCCTCC-3′,
- GAPDH, forward 5′- TTGGTATCGTGGAAGGACTCA-3′,
- GAPDH, reverse 5′- TGTCATCATATTTGGCAGGTT-3'.
- The mRNA levels of SMAD4 were normalized to GAPDH.

### Western blotting, immunoprecipitation, and sWGA affinity purification

Cells were washed with ice-cold PBS 3 times and lysed with NET lysis buffer. The buffer was composed of 150 mM NaCl, 50 mM Tris, 1 mM EDTA, and 1% Nonidet P-40, had a pH of 7.4, and was supplemented with a protease inhibitor cocktail (Roche). Lysates were boiled in the SDS sample buffer for 5 min and then subjected to Western blot analysis. The following antibodies were used:

Anti-*O*-GlcNAc (RL2: Thermo Fisher Scientific, #MA1-072), Anti-SMAD4 (Santa Cruz, #sc-7966), Anti-SMAD2/3 (Cell Signaling Technology, #3102S), Anti-OGT (DM-17: Sigma, #O6264), Anti-OGA (Abcam, #ab124807), Anti-GAPDH (Millipore, #M171-3 or Santa Cruz, #sc-32233), Anti-FLAG (MBL, #M185-3L), Anti-Ubiquitin (Santa Cruz, #sc-8017), Anti-HA (Santa Cruz, #sc-805 or Cell Signaling Technology, #C29F4), Anti-GSK-3β (Cell Signaling Technology, #27C10), Anti-Phospho-GSK-3β (Ser9) (Cell Signaling Technology, #D3A4), Anti-Phospho-GSK-3β (Tyr216) (Abcam, #ab75745), Anti-Phospho-threonine (Santa Cruz, #sc-5267), Anti-c-myc (Santa Cruz, #sc-47694).

For immunoprecipitation and sWGA purification, total cell lysates were incubated with antibody-bound beads or agarose-conjugated succinylated wheat germ agglutinin (sWGA, Vector Laboratories) overnight at 4 °C. Beads were then washed 5 times with lysis buffer and bead-bound proteins were eluted by boiling the beads for 5 min in the SDS sample buffer.

### Ubiquitination assay

HEK293 cells were transiently co-expressed with FLAG-SMAD4 and HA-ubiquitin. After 48 h of transfection, cells were incubated in 20 μM MG132 for 6 h and lysed with RIPA buffer. The buffer was composed of 150 mM NaCl, 50 mM Tris, 2 mM EDTA, 0.5% sodium deoxycholate, 50 mM NaF, 1% Nonidet P-40, and 0.1% SDS with a pH of 7.4. Total cell extracts were subjected to immunoprecipitation using FLAG antibody-bound A/G beads under denaturing conditions containing 2% SDS. The ubiquitination levels of SMAD4 were measured by Western blot analysis.

### Reporter gene assay

The reporter construct for (CAGA)_12_-Luc was kindly provided by Prof. Seong-Jin Kim (Seoul National University, Suwon, South Korea). MDA-MB-468 SMAD4 WT or T63V cells were transiently co-transfected with 0.5 μg of (CAGA)_12_-Luc and 0.02 μg of CMV-Renilla. Reporter gene activities were analyzed with the Dual-Luciferase Reporter Assay System (Promega) and normalized by renilla.

## Supplementary information

Supplementary Information 1.

Supplementary Information 2.

## Abbreviations

TGF-β: Transforming growth factor-β
EMT: Epithelial-mesenchymal transition
OGT: *O*-GlcNAc transferase
OGA: *O*-GlcNAcase
CHX: Cycloheximide
sWGA: Succinylated wheat germ agglutinin

## References

[1] Fuxe J, Vincent T, Garcia de Herreros A (2010). Transcriptional crosstalk between TGF-β and stem cell pathways in tumor cell invasion: Role of EMT promoting Smad complexes. Cell Cycle.

[2] Zarzynska JM (2014). Two faces of TGF-beta1 in breast cancer. Mediat. Inflamm..

[3] Principe DR (2014). TGF-β: Duality of function between tumor prevention and carcinogenesis. J. Natl. Cancer Inst..

[4] Lin HY, Wang XF, Ng-Eaton E, Weinberg RA, Lodish HF (1992). Expression cloning of the TGF-beta type II receptor, a functional transmembrane serine/threonine kinase. Cell.

[5] Lin HY, Moustakas A (1994). TGF-beta receptors: Structure and function. Cell Mol. Biol. (Noisy-le-grand).

[6] Ventura F, Doody J, Liu F, Wrana JL, Massagué J (1994). Reconstitution and transphosphorylation of TGF-beta receptor complexes. Embo J..

[7] Abdollah S (1997). TbetaRI phosphorylation of Smad2 on Ser465 and Ser467 is required for Smad2-Smad4 complex formation and signaling. J. Biol. Chem..

[8] Souchelnytskyi S (1997). Phosphorylation of Ser465 and Ser467 in the C terminus of Smad2 mediates interaction with Smad4 and is required for transforming growth factor-beta signaling. J. Biol. Chem..

[9] Zhang Y, Feng X, We R, Derynck R (1996). Receptor-associated Mad homologues synergize as effectors of the TGF-beta response. Nature.

[10] Macías-Silva M (1996). MADR2 is a substrate of the TGFbeta receptor and its phosphorylation is required for nuclear accumulation and signaling. Cell.

[11] Roberts AB, Wakefield LM (2003). The two faces of transforming growth factor beta in carcinogenesis. Proc. Natl. Acad. Sci U. S. A..

[12] Hannon GJ, Beach D (1994). p15INK4B is a potential effector of TGF-beta-induced cell cycle arrest. Nature.

[13] Li CY, Suardet L, Little JB (1995). Potential role of WAF1/Cip1/p21 as a mediator of TGF-beta cytoinhibitory effect. J. Biol. Chem..

[14] Fernandez-Pol JA, Talkad VD, Klos DJ, Hamilton PD (1987). Suppression of the EGF-dependent induction of c-myc proto-oncogene expression by transforming growth factor beta in a human breast carcinoma cell line. Biochem. Biophys. Res. Commun..

[15] Ling MT, Wang X, Tsao SW, Wong YC (2002). Down-regulation of Id-1 expression is associated with TGF beta 1-induced growth arrest in prostate epithelial cells. Biochim. Biophys. Acta.

[16] Thiery JP (2002). Epithelial-mesenchymal transitions in tumour progression. Nat. Rev. Cancer.

[17] Xu J, Lamouille S, Derynck R (2009). TGF-beta-induced epithelial to mesenchymal transition. Cell Res..

[18] Levy L, Hill CS (2005). Smad4 dependency defines two classes of transforming growth factor beta (TGF-{beta}) target genes and distinguishes TGF-{beta}-induced epithelial-mesenchymal transition from its antiproliferative and migratory responses. Mol. Cell Biol..

[19] Lamouille S, Xu J, Derynck R (2014). Molecular mechanisms of epithelial-mesenchymal transition. Nat. Rev. Mol. Cell Biol..

[20] Inman GJ (2011). Switching TGFβ from a tumor suppressor to a tumor promoter. Curr. Opin. Genet. Dev..

[21] Holt GD, Hart GW (1986). 1986 The subcellular distribution of terminal *N*-acetylglucosamine moieties. Localization of a novel protein-saccharide linkage, O-linked GlcNAc. J. Biol. Chem..

[22] Holt GD, Haltiwanger RS, Torres CR, Hart GW (1987). Erythrocytes contain cytoplasmic glycoproteins. O-linked GlcNAc on Band 41. J. Biol. Chem..

[23] Arvanitis DL, Arvanitis LD, Panourias IG, Kitsoulis P, Kanavaros P (2005). Mitochondria-rich normal, metaplastic, and neoplastic cells show overexpression of the epitope H recognized by the monoclonal antibody H. Pathol. Res. Pract..

[24] Marshall S, Bacote V, Traxinger RR (1991). Discovery of a metabolic pathway mediating glucose-induced desensitization of the glucose transport system. Role of hexosamine biosynthesis in the induction of insulin resistance. J. Biol. Chem..

[25] McClain DA, Crook ED (1996). Hexosamines and insulin resistance. Diabetes.

[26] Vosseller K, Wells L, Hart GW (2001). Nucleocytoplasmic *O*-glycosylation: *O*-GlcNAc and functional proteomics. Biochimie.

[27] Zachara, N., Akimoto, Y. & Hart, G. W. In *Essentials of Glycobiology* (eds A. Varki *et al.*) 239–251 (Cold Spring Harbor Laboratory Press Copyright 2015–2017 by The Consortium of Glycobiology Editors, La Jolla, California. All rights reserved., 2015).

[28] Wells L, Whelan SA, Hart GW (2003). *O*-GlcNAc: A regulatory post-translational modification. Biochem. Biophys. Res. Commun..

[29] Hart GW, Housley MP, Slawson C (2007). Cycling of O-linked beta-*N*-acetylglucosamine on nucleocytoplasmic proteins. Nature.

[30] Love DC, Hanover JA (2005). The hexosamine signaling pathway: Deciphering the "*O*-GlcNAc code". Sci. STKE.

[31] Fardini Y, Dehennaut V, Lefebvre T, Issad T (2013). *O*-GlcNAcylation: A new cancer hallmark?. Front. Endocrinol. (Lausanne).

[32] Slawson C, Hart GW (2011). *O*-GlcNAc signalling: Implications for cancer cell biology. Nat. Rev. Cancer.

[33] de Queiroz RM, Carvalho E, Dias WB (2014). *O*-GlcNAcylation: The sweet side of the cancer. Front. Oncol..

[34] Lynch TP (2012). Critical role of O-Linked β-*N*-acetylglucosamine transferase in prostate cancer invasion, angiogenesis, and metastasis. J. Biol. Chem..

[35] Caldwell SA (2010). Nutrient sensor *O*-GlcNAc transferase regulates breast cancer tumorigenesis through targeting of the oncogenic transcription factor FoxM1. Oncogene.

[36] Aguilar H (2014). Role for high-glucose-induced protein *O*-GlcNAcylation in stimulating cardiac fibroblast collagen synthesis. Am. J. Physiol. Cell Physiol..

[37] Tang X (2017). SIRT7 antagonizes TGF-β signaling and inhibits breast cancer metastasis. Nat. Commun..

[38] Demagny H, Araki T, De Robertis EM (2014). The tumor suppressor Smad4/DPC4 is regulated by phosphorylations that integrate FGF, Wnt, and TGF-β signaling. Cell Rep..

[39] Zhang F (2003). *O*-GlcNAc modification is an endogenous inhibitor of the proteasome. Cell.

[40] van der Laarse SAM, Leney AC, Heck AJR (2018). Crosstalk between phosphorylation and *O*-GlcNAcylation: Friend or foe. Febs J..

[41] Maury JJ, Ng D, Bi X, Bardor M, Choo AB (2014). Multiple reaction monitoring mass spectrometry for the discovery and quantification of *O*-GlcNAc-modified proteins. Anal. Chem..

[42] Krishnankutty A (2017). In vivo regulation of glycogen synthase kinase 3β activity in neurons and brains. Sci. Rep..

[43] Wan M (2004). Smad4 protein stability is regulated by ubiquitin ligase SCF beta-TrCP1. J. Biol. Chem..

[44] Drabsch Y, ten Dijke P (2012). TGF-β signalling and its role in cancer progression and metastasis. Cancer Metastasis Rev..

[45] Ma Z, Vosseller K (2013). *O*-GlcNAc in cancer biology. Amino Acids.

[46] Vander Heiden MG, Cantley LC, Thompson CB (2009). Understanding the Warburg effect: The metabolic requirements of cell proliferation. Science.

[47] Lucena MC (2016). Epithelial mesenchymal transition induces aberrant glycosylation through hexosamine biosynthetic pathway activation. J. Biol. Chem..

[48] Duan F (2018). *O*-GlcNAcylation of RACK1 promotes hepatocellular carcinogenesis. J. Hepatol..

[49] Li Y (2017). *O*-GlcNAcylation modulates Bmi-1 protein stability and potential oncogenic function in prostate cancer. Oncogene.

[50] Li MD (2013). *O*-GlcNAc signaling entrains the circadian clock by inhibiting BMAL1/CLOCK ubiquitination. Cell Metab..

[51] Ferrer CM (2014). O-GlcNAcylation regulates cancer metabolism and survival stress signaling via regulation of the HIF-1 pathway. Mol. Cell.

[52] Park SY (2010). Snail1 is stabilized by *O*-GlcNAc modification in hyperglycaemic condition. Embo J..

[53] Yang WH (2006). Modification of p53 with O-linked *N*-acetylglucosamine regulates p53 activity and stability. Nat. Cell Biol..

[54] Sayat R, Leber B, Grubac V, Wiltshire L, Persad S (2008). *O*-GlcNAc-glycosylation of beta-catenin regulates its nuclear localization and transcriptional activity. Exp. Cell Res..

[55] Chou TY, Dang CV, Hart GW (1995). Glycosylation of the c-Myc transactivation domain. Proc. Natl. Acad. Sci. U. S. A..

[56] Zhou BP (2004). Dual regulation of Snail by GSK-3beta-mediated phosphorylation in control of epithelial-mesenchymal transition. Nat. Cell Biol..

[57] Olivier-Van Stichelen S (2014). *O*-GlcNAcylation stabilizes β-catenin through direct competition with phosphorylation at threonine 41. Faseb J..

[58] Liu C (1999). beta-Trcp couples beta-catenin phosphorylation-degradation and regulates Xenopus axis formation. Proc. Natl. Acad. Sci. U. S. A..

[59] Zeng Y (2016). Repression of Smad4 by miR-205 moderates TGF-β-induced epithelial-mesenchymal transition in A549 cell lines. Int J Oncol.

