# Supplementary material for: O-GlcNAc stabilizes SMAD4 by inhibiting GSK-3β-mediated proteasomal degradation
Source: Sci Rep. 2020 Nov 16;10:19908. doi: 10.1038/s41598-020-76862-0 (PMC7670456; doi:10.1038/s41598-020-76862-0)
Supplement: Supplementary file 1 — Supplementary Information 1. [file 41598_2020_76862_MOESM1_ESM.docx]

**Supplementary informations**

***O*-GlcNAc stabilizes SMAD4 by inhibiting GSK-3β-mediated proteasomal degradation**

**Yeon Jung Kim^1^, Min Jueng Kang^4^, Eunah Kim^1^, Tae Hyun Kweon^1, 3^, Yun Soo Park^1, 3^, Suena Ji^1^, Won Ho Yang^1, 2^, Eugene C. Yi^1, 4^ and Jin Won Cho^1, 2, 3, *^**

^1^Glycosylation Network Research Center, Yonsei University, 50 Yonsei-ro, Seodaemun-gu, Seoul 03722, Republic of Korea

^2^Department of Systems Biology, College of Life Science and Biotechnology, Yonsei University, 50 Yonsei-ro, Seodaemun-gu, Seoul 03722, Republic of Korea

^3^Interdisciplinary Program of Integrated OMICS for Biomedical Science, Graduate School, Yonsei University, 50 Yonsei-ro, Seodaemun-gu, Seoul 03722, Republic of Korea

^4^Department of Molecular Medicine and Biopharmaceutical Sciences, School of Convergence Science and Technology and College of Medicine or College of Pharmacy, Seoul National University, 28 Yeongeon-dong, Jongno-gu, Seoul 03080, Republic of Korea

^*^[chojw311@yonsei.ac.kr](mailto:chojw311@yonsei.ac.kr)

**
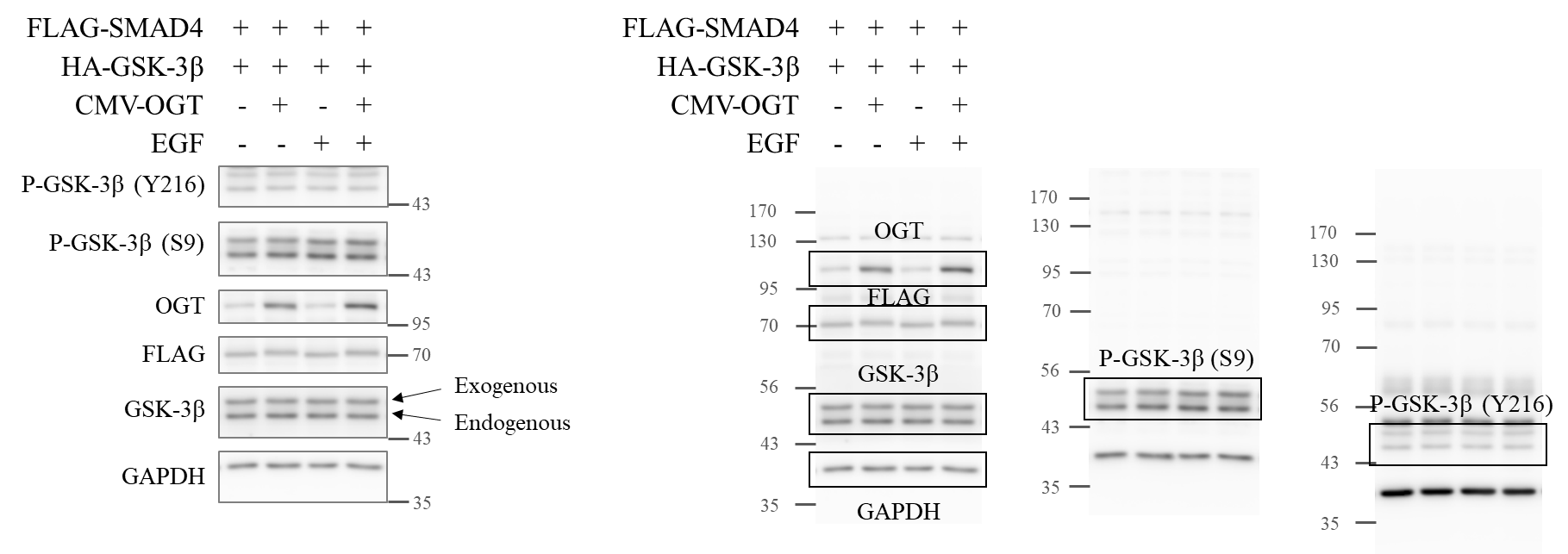
**

**Supplementary Figure S1.** Immunoblots showing no significant changes in GSK-3β activity upon OGT overexpression and its uncropped immunoblots. HEK293 cells were prepared as indicated in Fig. 2F.

**
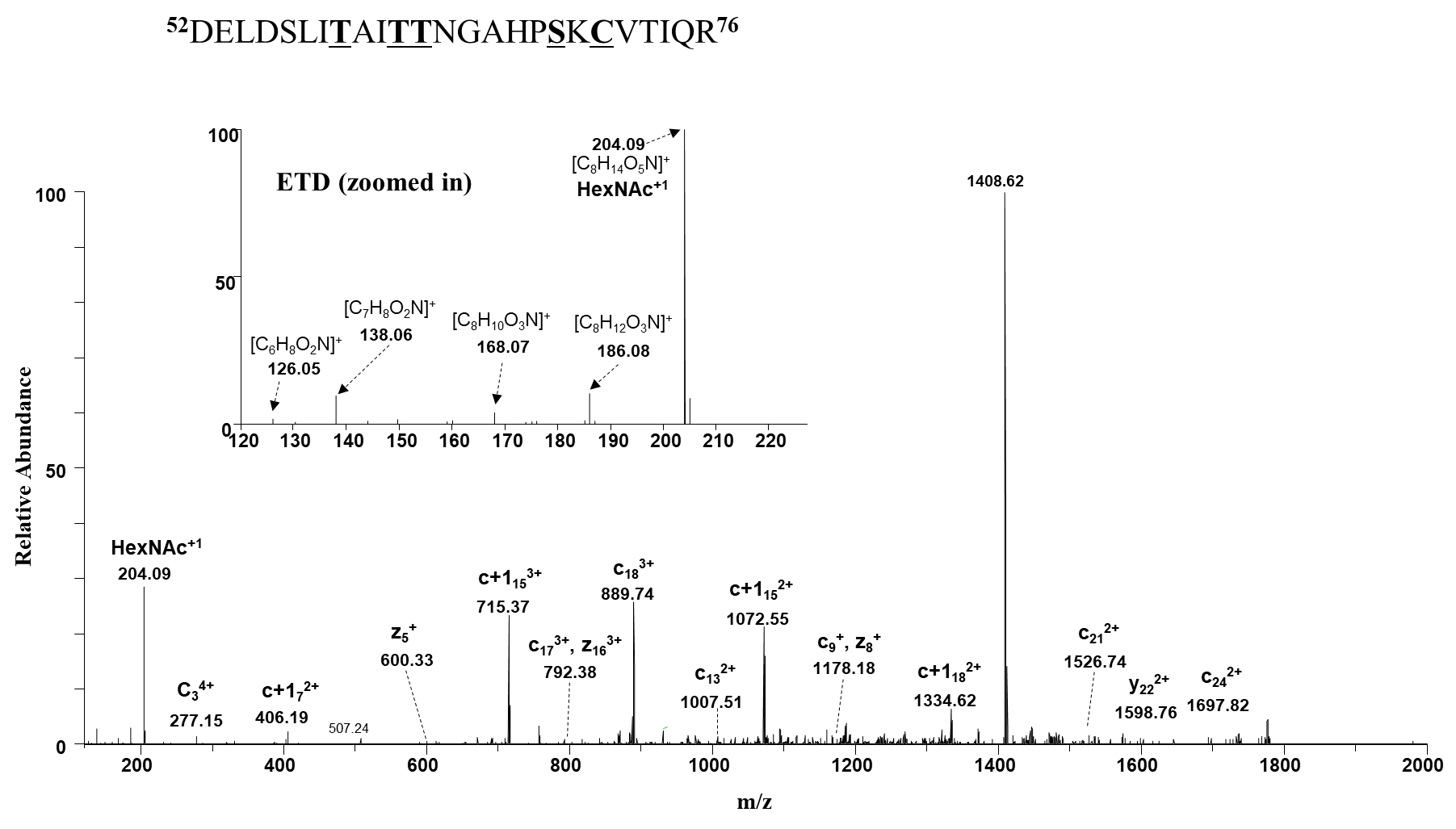
Supplementary Figure S2.** Mass spectrometry analysis showing *O*-GlcNAc modification within SMAD4. The ETD MS/MS spectrum of an *O*-GlcNAcylated peptide of SMAD4 (residues 52–76) with the quadruply charged precursor ion m/z 888.93 is shown. The c- and z-type product ions were assigned. The *O*-GlcNAc oxonium ion (204.09 m/z) and a series of its fragments (126.05, 138.05, 168.0, and 186.08 m/z) were also assigned.

**
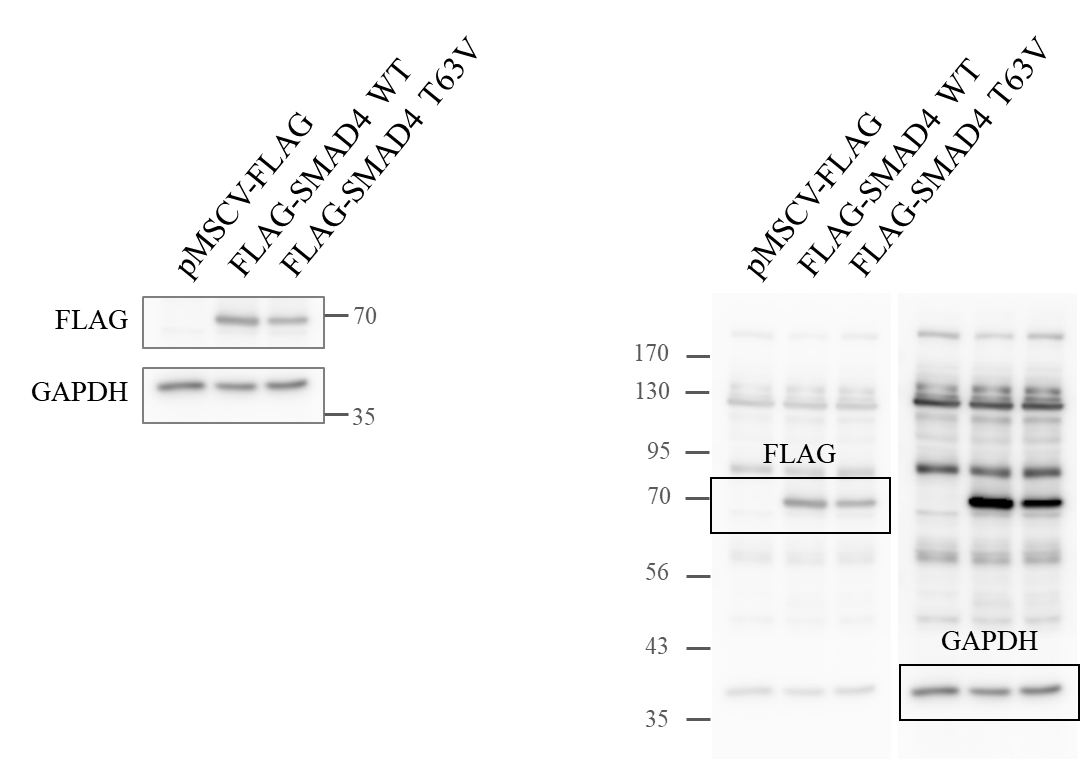
**

**Supplementary Figure S3.** Immunoblots showing FLAG-SMAD4 WT and T63V overexpression in SMAD4^-/-^ MDA-MB-468 cells and its uncropped immunoblots. Cells were established by introducing retroviral particles from HEK293FT cells.


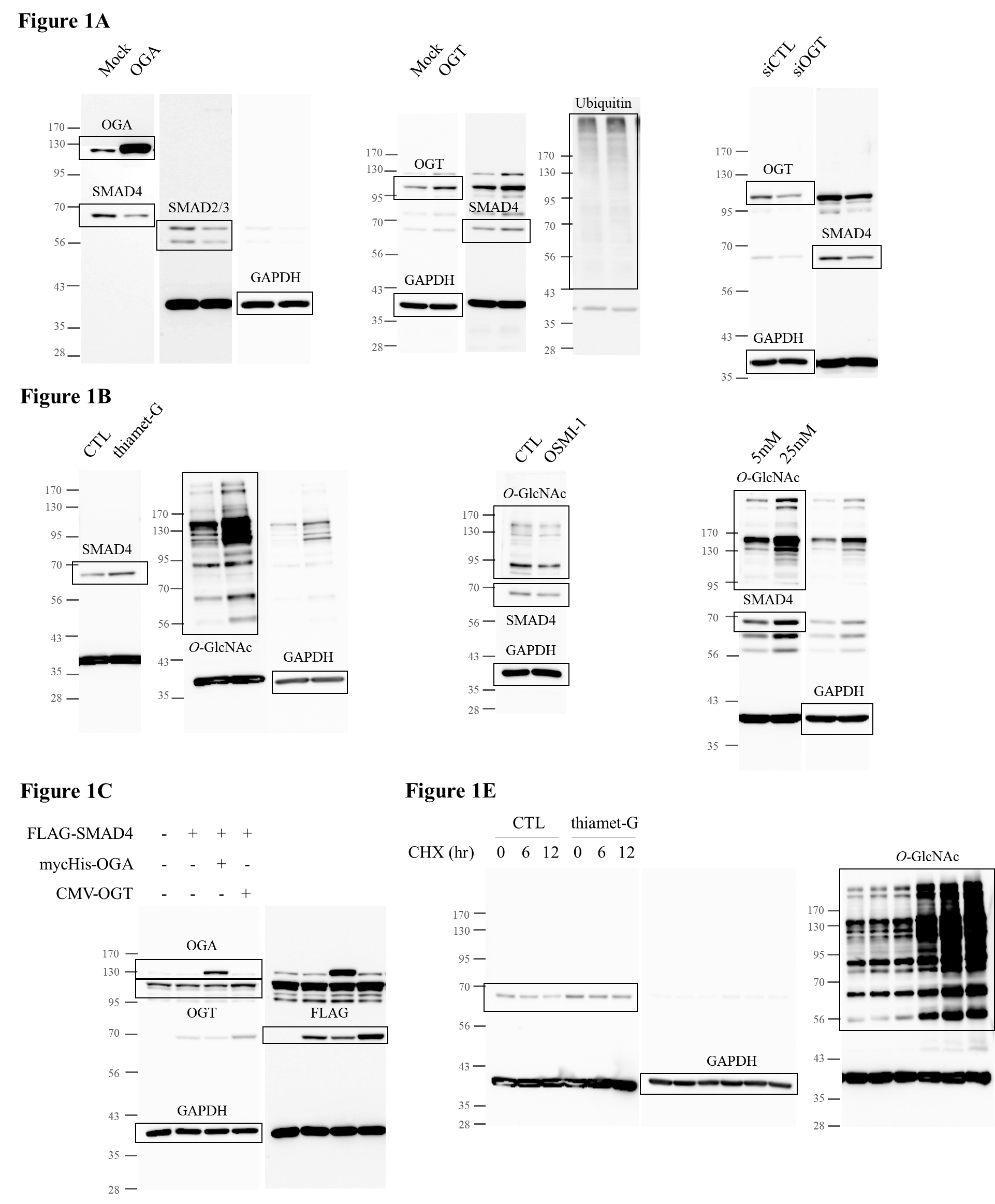


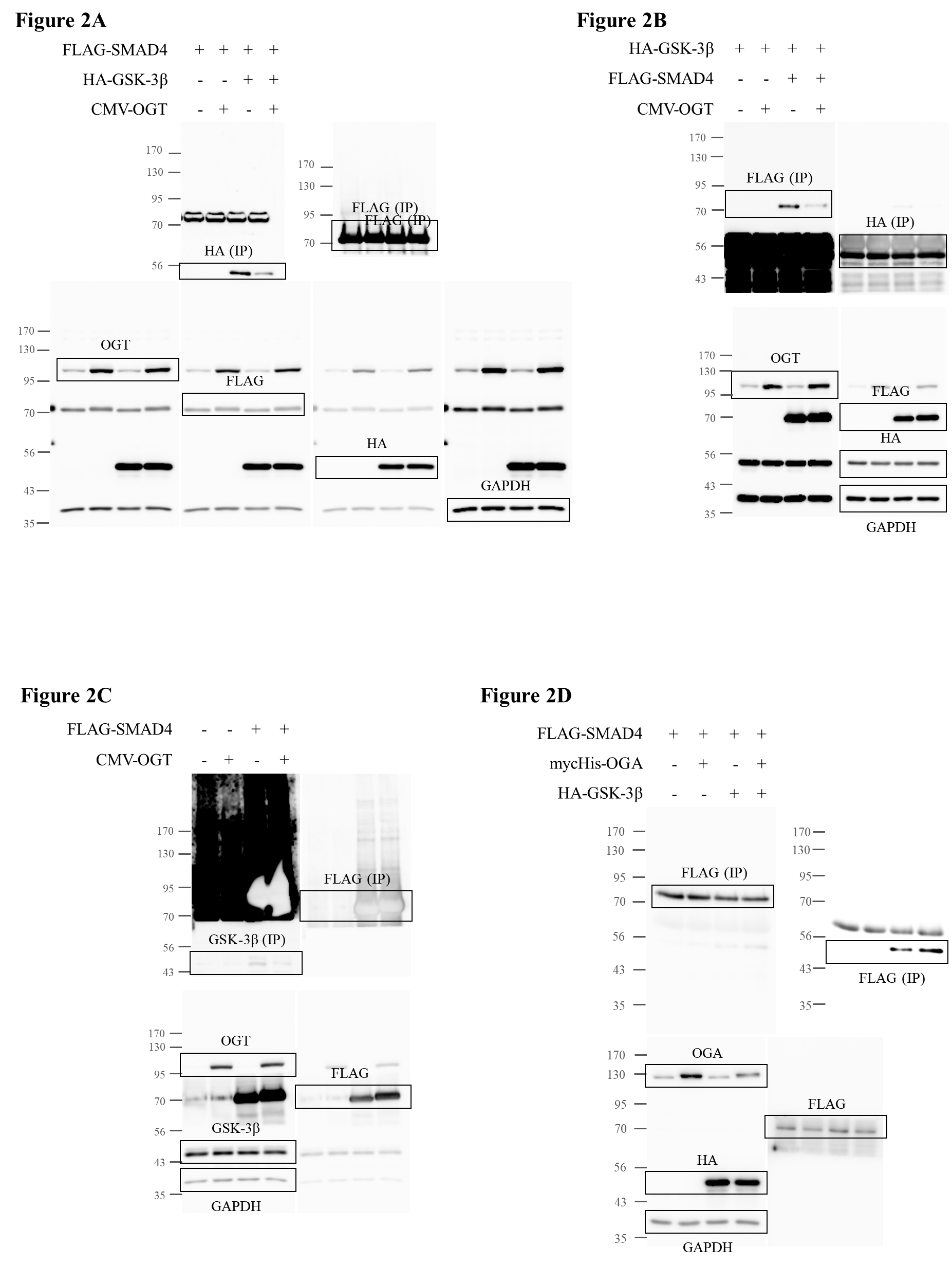


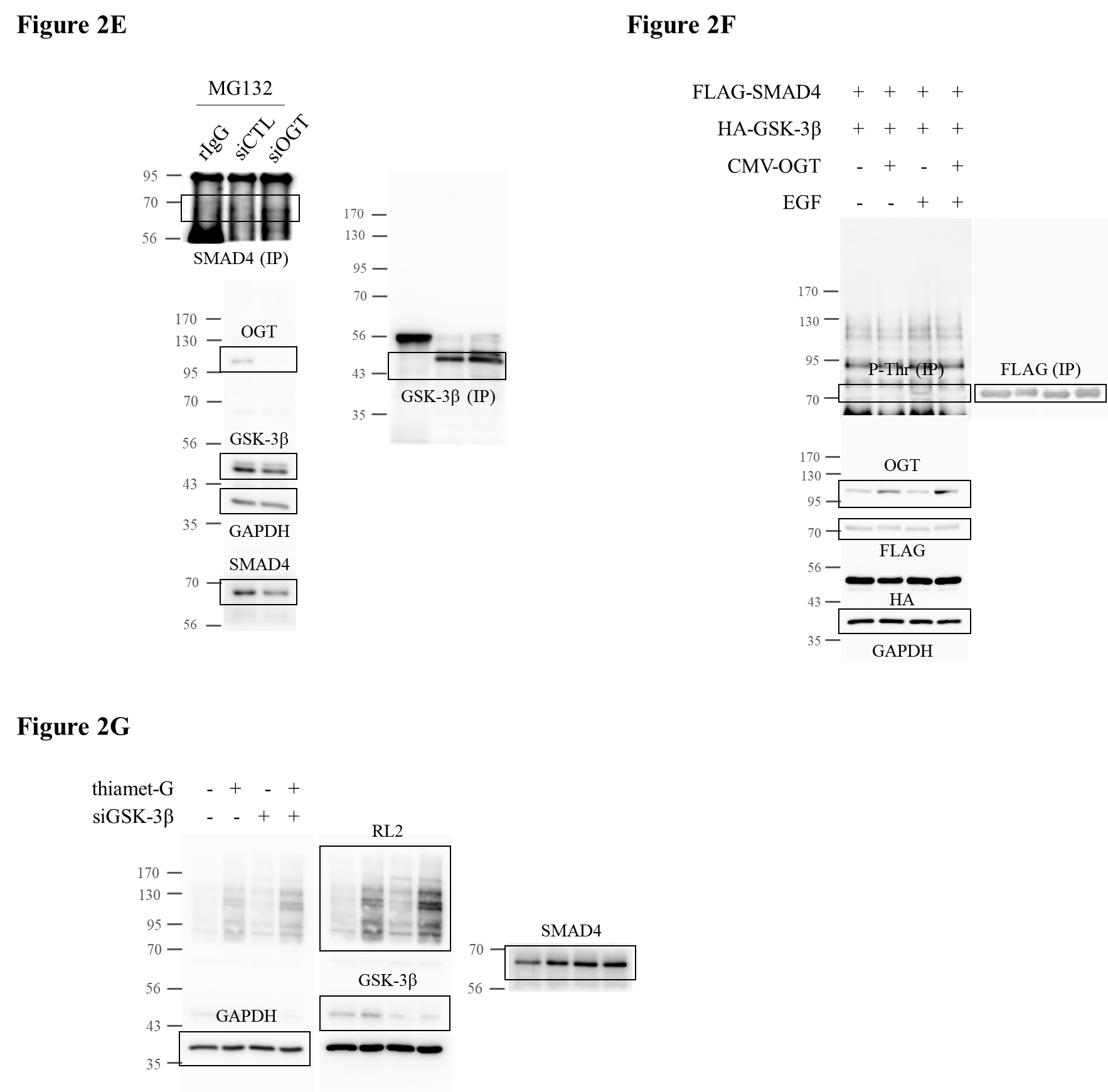


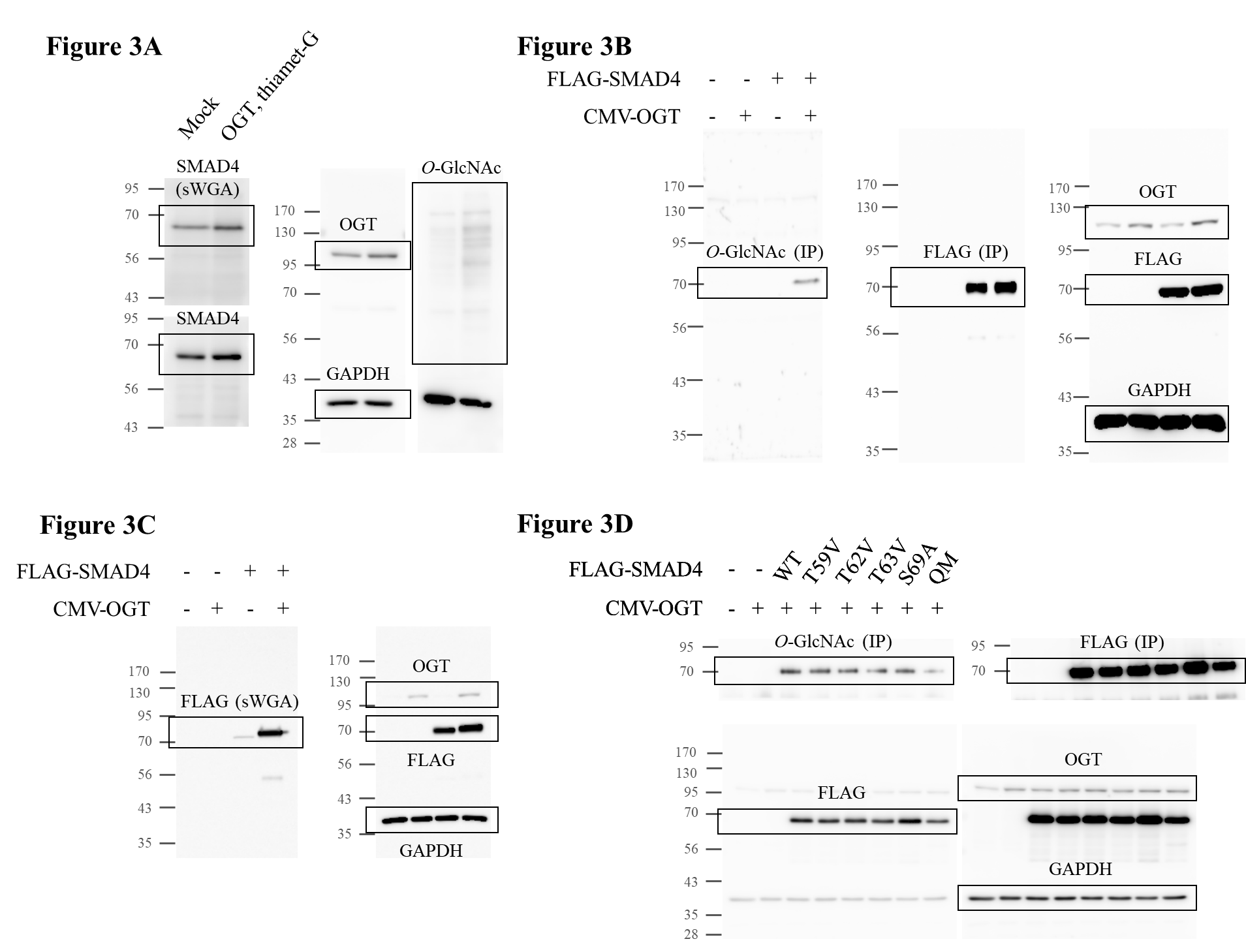


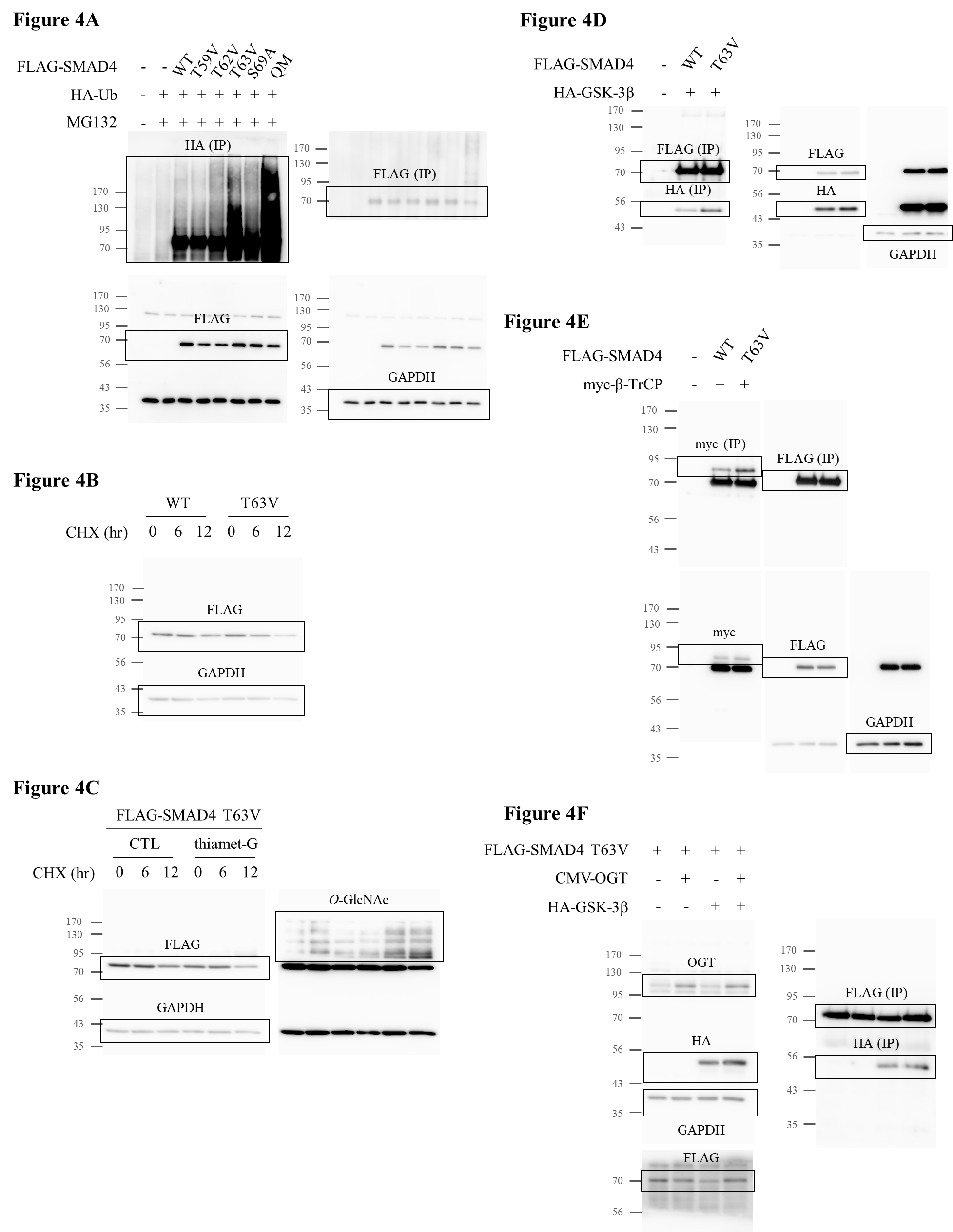


**Supplementary Figure S4.** Uncropped immunoblots, corresponding to the indicated figures in the

manuscript.
