# Supplementary material for: O-GlcNAc stabilizes SMAD4 by inhibiting GSK-3β-mediated proteasomal degradation
Source: Sci Rep. 2020 Nov 16;10:19908. doi: 10.1038/s41598-020-76862-0 (PMC7670456; doi:10.1038/s41598-020-76862-0)
Supplement: Supplementary file 2 — Supplementary Information 2. [file 41598_2020_76862_MOESM2_ESM.docx]

**Supplementary Tables**

***O*-GlcNAc stabilizes SMAD4 by inhibiting GSK-3β-mediated proteasomal degradation**

**Yeon Jung Kim^1^, Min Jueng Kang^4^, Eunah Kim^1^, Tae Hyun Kweon^1, 3^, Yun Soo Park^1, 3^, Suena Ji^1^, Won Ho Yang^1, 2^, Eugene C. Yi^1, 4^ and Jin Won Cho^1, 2, 3, *^**

^1^Glycosylation Network Research Center, Yonsei University, 50 Yonsei-ro, Seodaemun-gu, Seoul 03722, Republic of Korea

^2^Department of Systems Biology, College of Life Science and Biotechnology, Yonsei University, 50 Yonsei-ro, Seodaemun-gu, Seoul 03722, Republic of Korea

^3^Interdisciplinary Program of Integrated OMICS for Biomedical Science, Graduate School, Yonsei University, 50 Yonsei-ro, Seodaemun-gu, Seoul 03722, Republic of Korea

^4^Department of Molecular Medicine and Biopharmaceutical Sciences, School of Convergence Science and Technology and College of Medicine or College of Pharmacy, Seoul National University, 28 Yeongeon-dong, Jongno-gu, Seoul 03080, Republic of Korea

^*^[chojw311@yonsei.ac.kr](mailto:chojw311@yonsei.ac.kr)

**
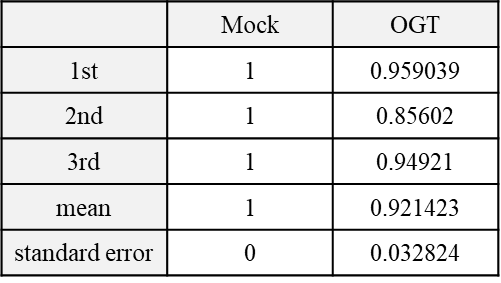
**

**Supplementary Table S1.** Data used for statistical analysis, corresponding to the figure 1D in the manuscript.

**
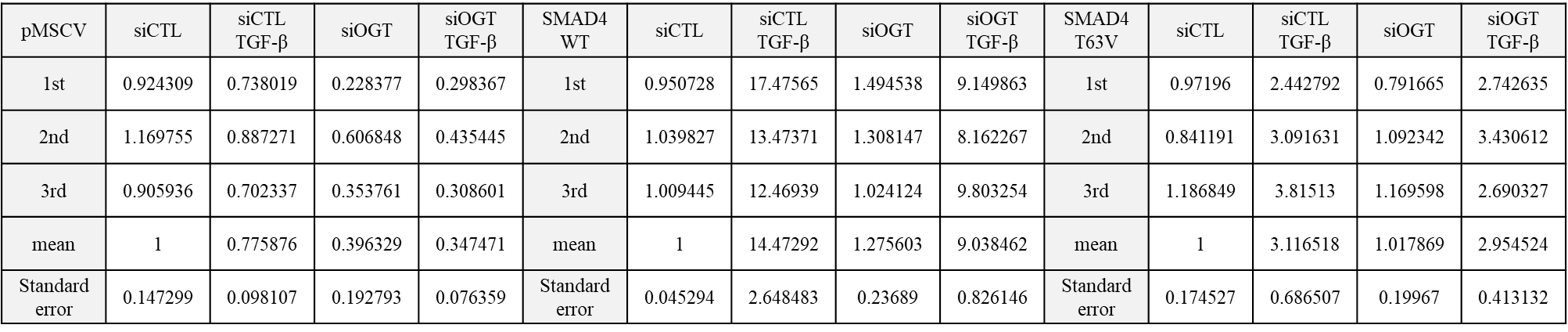
**

**Supplementary Table S2.** Data used for statistical analysis, corresponding to the figure 5A in the manuscript.
